# Evolution of CG Methylation Maintenance Machinery in Plants

**DOI:** 10.3390/epigenomes5030019

**Published:** 2021-09-14

**Authors:** Louis Tirot, Pauline E. Jullien, Mathieu Ingouff

**Affiliations:** 1 IPS, University of Bern, 3013 Bern, Switzerland; louis.tirot@ips.unibe.ch; 2 CIRAD, DIADE, IRD, University of Montpellier, 34393 Montpellier, France

**Keywords:** DNA methylation, DNA METHYLTRANSFERASE 1, MET1, epigenetics, plant

## Abstract

Cytosine methylation is an epigenetic mark present in most eukaryotic genomes that contributes to the regulation of gene expression and the maintenance of genome stability. DNA methylation mostly occurs at CG sequences, where it is initially deposited by de novo DNA methyltransferases and propagated by maintenance DNA methyltransferases (DNMT) during DNA replication. In this review, we first summarize the mechanisms maintaining CG methylation in mammals that involve the DNA Methyltransferase 1 (DNMT1) enzyme and its cofactor, UHRF1 (Ubiquitin-like with PHD and RING Finger domain 1). We then discuss the evolutionary conservation and diversification of these two core factors in the plant kingdom and speculate on potential functions of novel homologues typically observed in land plants but not in mammals.

## 1. Introduction

DNA methylation is a highly conserved DNA modification, present across eukaryotes of the plant and animal kingdoms [1,2,3,4]. However, it is not universal, as certain non-plant eukaryotic genomes are devoid of DNA methylation [5,6]. DNA methylation is a covalent DNA modification affecting cytosine residues. It is typically involved in the regulation of gene expression and the silencing of transposable elements (TEs), by which it ensures genomic stability. In addition, DNA methylation is central to developmental processes such as genomic imprinting and X-chromosome inactivation [7,8].

Although DNA methylation occurs in CG and non-CG sites (CH, where H = A, T or C) in both mammals and plants, these two types of DNA methylation vary in terms of their genomic distribution and occurrence during development [3,9,10] as well as their dedicated enzymatic machinery [2]. In mammals, CG methylation is the main type of DNA methylation, and it covers the bodies of most genes and TEs [9,10,11,12]. In plants, CG methylation is detected only on a limited set of genes and TEs are covered by both CG and non-CG methylation [1,5,13,14]. CG methylation is ubiquitously detected during both plant and mammalian life cycle. However, in contrast to plants, non-CG methylation is only detected in specific mammalian tissues or cell types [15].

The establishment of a new DNA methylation pattern or de novo DNA methylation corresponds to the addition of a methyl group at the position C5 of an unmodified cytosine residue (5mC). As de novo methylation generates new DNA methylation patterns, the corresponding enzymatic machineries need to be highly regulated and precisely targeted. In mammals, de novo DNA methylation involves the de novo DNA methyltransferases 3 (DNMT3). DNMT3 enzymes methylate cytosine residues in all sequence contexts and are targeted by direct interaction with histone post-translational marks [16,17]. In contrast, de novo DNA methylation in plants involves the RNA-dependent DNA methylation (RdDM) pathway, which targets the DOMAINS REARRANGED METHYLTRANSFERASE 1 and 2 (DRM1, DRM2) to cytosines, guided by small RNA molecules [1,18,19].

After the establishment of novel DNA methylation marks, the newly created patterns must be faithfully transmitted by maintenance DNA methyltransferases during cell division [20,21]. CG methylation is maintained by two evolutionarily conserved core partners: (1) a maintenance DNA methyltransferase called DNMT1 in mammals and MET1 (DNA METHYLTRANSFERASE 1) in plants and (2) a cofactor named UHRF1 in mammals and VIM (VARIANT IN METHYLATION) in plants [2]. In mammals, the maintenance of non-CG methylation typically involves DNMT3 enzymes [15]. In plants, non-CG methylation is further divided in two classes of sequence—CHG and CHH [1,3,13,22]—and requires distinct enzymatic machineries. The maintenance of CHG sites relies on the plant-specific CHROMOMETHYLASE3 (CMT3) in cooperation with H3K9 histone methyltransferases [23,24,25,26,27,28]. Maintenance of the CHH context requires the combined action of the CHROMOMETHYLASE2 (CMT2) and the de novo methylation machinery, i.e., the RdDM pathway [26,27]. DNA methylation patterns can rapidly be lost by both passive and active DNA demethylation. Passive demethylation results from the absence of the recruitment of DNA methyltransferases during DNA replication while active DNA demethylation requires specific enzymes that differ between plants and mammals. In plants, active DNA demethylation is driven by DNA glycosylases that excise 5mC in all sequence contexts [29,30]. In mammals, ten-eleven translocation (TET) methylcytosine dioxygenases catalyze the conversion of 5mC to 5hmC (5-hydroxymethylcytosines) and further oxidation products. These modified cytosines can be retained or ultimately be replaced by naive cytosines [17,31,32].

As several recent reviews on non-CG methylation machineries and their evolution in plants are available [19,33,34,35], we focus here on the core actors of the maintenance of CG methylation. We first summarize the molecular mechanisms of the maintenance CG methylation in mammals and further discuss its conservation in plants. We then evaluate the diversification of the central actors in this process during plant evolution. Finally, we speculate on the potential roles of recently diversified factors in higher plants.

## 2. Molecular Mechanisms of the DNMT1/UHRF1 Pathway

**Figure 1:**
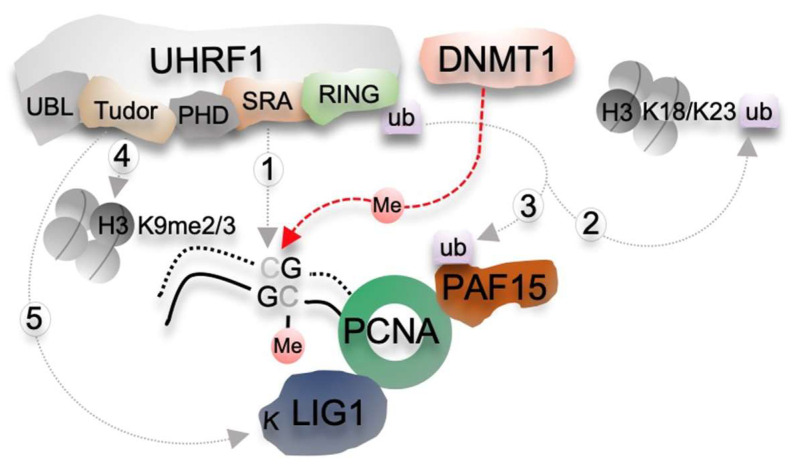
Molecular mechanisms of CG methylation maintenance in mammals. CG methylation maintenance involves the maintenance DNA methyltransferase DNMT1 and distinct functional domains of UHRF1. UHRF1 is targeted to hemi-methylated DNA formed after DNA replication through its SRA domain (1) and ubiquitylates (ub) lysine (K) residues either on PAF15 (PCNA-associated factor 15) (2) or on histone H3 (3) through its E3 ubiquitin ligase RING domain. DNMT1 recognizes these ubiquitinated residues via its RFTS domain and restores symmetric CG methylation. In addition, The Tudor domain of UHRF1 binds H3K9me3 histone mark (4) and a methylated histone-like motif in DNA ligase 1 (K-LIG1) (5) enzyme that joins Okazaki fragments generated in the lagging strands. These interactions further facilitate the maintenance of CG methylation. Abbreviations: Me, methylated; PHD, Plant Homeodomain finger; RING, Really Interesting New Gene domain; SRA, su(var)3-9, enhancer-of-zeste–trithorax (SET) and RING-associated domain; TTD, Tudor domain; UBL, Ubiquitin-like domain.

During DNA replication, the parental DNA methylation pattern needs to be copied to newly synthesized daughter strands, which are devoid of DNA methylation. In mammals, multiple DNA replication-coupled methylation maintenance pathways are at play to faithfully propagate CG methylation throughout the genome and involve two main players: the DNMT1 enzyme and its cofactor UHRF1 (Figure 1) [36,37,38].

**Figure 2:**
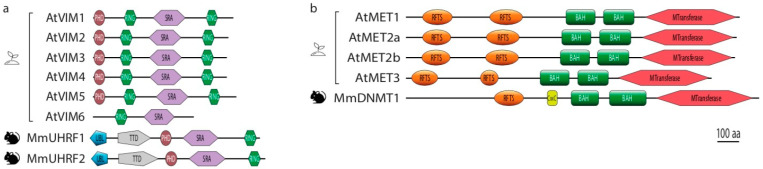
Domain structures of the core players of CG methylation maintenance in plants (Arabidopsis) and mammals (mouse). (**a**). Domains identified in DNMT1 DNA methyltransferase and corresponding homologs MET in Arabidopsis. (**b**). Domains identified in DNMT1 cofactor UHRF1 and their corresponding homologs VIM in Arabidopsis. Abbreviations: BAH, Bromodomain-adjacent homology domain; CXXC, CXXC Zinc-finger domain; MTransferase, methyltransferase domain; PHD, Plant Homeodomain finger; RING, Really Interesting New Gene domain; RFTS, Replication Foci Targeting Sequence domain; SRA, su(var)3-9, enhancer-of-zeste–trithorax (SET) and RING-associated domain; Tudor, Tudor domain; UBL, Ubiquitinlike domain.

DNMT1 is the main CG maintenance DNA methyltransferase in mammals. It is recruited concomitantly to DNA replication at hemi-methylated CG sites (hemi-mCG) to methylate the cytosines on the newly synthetized DNA strands and is therefore key to maintaining symmetrical CG methylation patterns. DNMT1 typically combines a N-terminal Replication Foci Targeting Sequence (RFTS) domain responsible for its targeting to the replication foci, a CXXC Zinc-finger domain, two bromodomain-adjacent homology (BAH) domains and a large C-terminal methyltransferase (MTase) domain (Figure 2b) [39,40,41].

Biochemical studies on DNMT1 revealed a higher efficiency on hemi-methylated targets compared to unmethylated targets therefore ensuring the proper maintenance of DNA methylation [42,43]. The de novo methylation activity of DNMT1 is prevented by two auto-inhibitory regulations: (1) an intramolecular interaction between the RFTS domain with the MTase catalytic domain locks DNMT1 methyltransferase activity until needed [44,45] and (2) the binding of the CXXC domain to unmethylated cytosines prevents the DNMT1 catalytic cleft from accessing these sequences [39,40].

The recruitment of DNMT1 to replicated sites can occur through an interaction with PCNA (Proliferating cell nuclear antigen) [46]. However, PCNA-binding deficient *dnmt1* mutants were still able to rescue *dnmt1* ES cells suggesting that PCNA-dependent recruitment of DNMT1 is not essential in maintaining DNA methylation [46]. DNMT1 recruitment and activation at hemi-mCG sequences is intimately linked to its cofactor UHRF1. *Uhrf1* loss-of-function leads to genome-wide demethylation as observed for *dnmt1* knock-out [47]. UHRF1 is a multidomain protein with a ubiquitin-like (UBL) domain, two adjacent histone reader domains, a Tudor domain (TTD) followed by a PHD (Plant Homeodomain) finger that recognize, respectively, di/trimethylated lysine 9 on histone3 (H3K9me2/3) and unmodified arginine on H3 (H3R2), a su(var)3-9, enhancer-of-zeste–trithorax (SET)- and RING-associated (SRA) domain and a Really Interesting New Gene (RING) E3 ubiquitin ligase domain (Figure 1 and Figure 2a). UHRF1 can directly interact and recruit DNMT1 to hemi-mCG via its SRA methyl-binding domain (Figure 1, arrow 1) [48,49].

Interestingly, UHRF1 also provides a link between the maintenance of DNA methylation and histone or histone-like modifications. Indeed, the UHRF1 RING domain mono-ubiquitylates lysine residues in histone H3 and a H3 mimic domain present in the DNA replication factor PAF15 (PCNA-associated factor 15) (Figure 1, arrow 2 and 3). Each of these two modifications is recognized by the RFTS domain of DNMT1 and contributes to the maintenance of CG methylation [50,51]. Additionally, a methylated histone H3K9 mimic domain lying within the DNA ligase 1 (LIG1)—an enzyme that joins nicks in the lagging strand—is recognized by the UHRF1 histone reader TTD domain that ultimately favors maintenance methylation (Figure 1, arrow 5) [52,53]. The UHRF1 TTD domain also recognizes H3K9me2/me3 histone marks (Figure 1, arrow 4) [54,55] and contributes to DNMT1 recruitment through its H3K9me RFTS reader domain to heterochromatin regions [16]. Altogether the different domains of UHRF1 are thus essential to recruit and activate DNMT1 at hemi-methylated CG DNA therefore ensuring the proper maintenance of DNA methylation during DNA replication.

## 3. Molecular Mechanisms of the MET/VIM Pathway in Plants

In both plant and animal genomes, the presence of DNMT1/UHRF1 homologues coincides with the detection of CG methylation [5,56]. For example, Drosophila and *C. elegans* genomes typically lack both cytosine methylation and *UHRF1* genes [3]. These observations suggest a conservation of core mechanisms involved in the maintenance of DNA methylation during evolution. DNMT1 and UHRF1 homologues have been identified in plants and are called MET1 and VIM, respectively. Similarly to mammals, mutations affecting those genes in Arabidopsis lead to a loss of CG methylation [9,57,58,59]. However, not enough is known at present in plants to conclude whether molecular mechanisms comparable to mammals (see above) are at play.

In Arabidopsis, the predicted MET proteins, including the functional MET1, share most of the domains present in mouse DNMT1 [60,61]. For example, all MET proteins in Arabidopsis have two RFTS domains (only one in DNMT1), two BAH domains and a C-terminal methyltransferase domain. The main difference is the absence of the CXXC domain in plant METs which, in DNMT1, reduces potential de novo activity [40]. Whether this activity is regulated for MET1 is currently unknown but a potential de novo activity of MET1 seems involved in de novo gene body methylation [62]. MET1 might, therefore, be more prone to induce de novo methylation than its mammalian counterpart due to the absence of the CXXC domain. Despite the presence of a conserved C-terminal methyltransferase domain in all Arabidopsis MET proteins, an enzymatic activity is only clear for MET1 and further experiments are needed to test whether MET1 paralogues have retained a functional methyltransferase activity.

In terms of domain structure, Arabidopsis VIMs show more differences than their mammalian counterpart, especially on their N-terminal part. They all have a N-terminal PHD domain and two RING domains flanking the SRA domain except VIM6 that lacks the PHD domain and C-terminal RING domain. Although each of the two RING domains of the tested VIM is sufficient to generate an E3 ligase activity [63] it is unclear whether VIM6 is still a functional enzyme. VIM proteins have retained most of the UHRF1 domains except the Tudor domain and the UBL domain localized on the N-terminal (Figure 2). The absence of Tudor domain in VIM proteins suggests a potential loss of a direct link between histone methylation and CG methylation maintenance. At present, biochemical analyses confirmed that all the Arabidopsis VIM tested have an E3 ubiquitin ligase activity [63,64] and preferentially bind to methylated CG in vitro but also to methylated CHG [25,65]. Some identified targets for ubiquitination by UHRF1 like LIG1 and histones H3 are well-conserved in plant genomes [66,67]. However, further experiments are needed to determine if these proteins are still targeted by VIM in plants.

## 4. Duplication of the MET and VIM Proteins in Plants

The mammalian genome (mouse and human) encodes only one *DNMT1* gene but two *UHRF* genes (*UHRF1, UHRF2)*. As both DNMT1 and UHRF1 are essential to maintain CG DNA methylation, *dnmt1* and *uhrf1* mutants suffer several defects and are embryo lethal. Interestingly, UHRF2 does not act redundantly with UHRF1 in maintaining CG methylation and *uhrf2* does not complement the *uhrf1* phenotype [41,68]. UHRF2 seems to be involved in cell cycle progression and possibly tumorigenesis via its binding to hydroxymethylated DNA [69,70]. This suggests that these two highly similar UHRF proteins have distinct functions in mammals.

**Figure 3:**
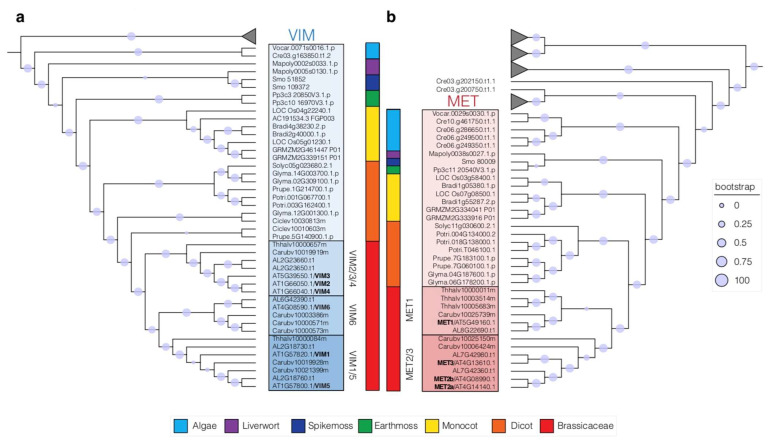
Phylogenetic trees inferring phylogenetic relationships of VIM cofactors (**a**) and MET (**b**) DNA methyltransferases in green lineages. VIM proteins are shaded in blue (**a**) and MET proteins are shaded in red (**b**). Bootstrap values are represented by circles and their corresponding legends. Branches corresponding to additional clades of SAD_SRA domain proteins or DNA methylase proteins were collapsed. Genomes used for the phylogenetic analyses: Algae (*C. reinhardtii*, *V. carteri*), Livewort (*M. polymorpha*)*,* Spikemoss (*S. moellendorfii*), Earthmoss (*P. patens*), Monocot (*B. distachyon*, *O. sativa*, *Z. mays*), Dicot (*C. clementine*, *G. max*, *P. trichocarpa, P. persica*, *S. lycopersicum*), Brassicacea *(A. lyrata*, *A. thaliana*, *C. rubella*, *E. salsugineum*). The full trees can be found in Figure S1 for the SAD_SRA domain proteins and in Figure S2 for the DNA methylase domain proteins.

To evaluate the degree of duplication as well as investigating the degree of conservation of MET and VIM protein in plants, we generated two phylogenetic trees (Figure 3a,b). We narrowed our analyses to a few well-annotated species representing major clades of the plant kingdom: *A. lyrata*, *A. thaliana*, *C. rubella*, *E. salsugineum*, *P. trichocarpa*, *G. max*, *S. lycopersicum*, *Z. mays*, *O. sativa*, *S. moellendorfii*, *P. patens*, *C. reinhardtii*, *V. carteri*, *M. polymorpha*, *C. clementine*, *P. persica*, *B. distachyon*. METs and VIMs protein sequences were obtained from the PHYTOZOME database [71] and filtered for the presence of specific PFAM protein domain: the C-5 cytosine-specific DNA methylase domain (PF00145) for MET proteins and the SET and Ring finger Associated, YDG motif protein domain (SAD_SRA, PF02182) for the VIM proteins. Details can be found in the legend of Figures S1 and S2.

The resulting trees show that both METs and VIMs clades are present in unicellular algae and have most likely be inherited from a common eukaryotic ancestor. Additionally, the SAD_SRA domain protein tree illustrates an early divergence of the VIM proteins with the other plant SAD_SRA proteins like other histone methyltransferases such as KRYPTONITE (KYP) proteins (H3K9 methyltransferases) (Figure S1). Similarly, the tree of plant proteins containing a DNA methylase domain shows a clear separation between the different classes of plant DNA methyltransferases: DRMs, CMTs and METs (Figure S2).

In our phylogenic analysis of UHRF homologues in plants, we can see that all plant genomes have at least one VIM protein (Figure 3a). In contrast to algae, which only possess one VIM protein, all other plants analyzed have at least two copies. The number of VIM homologs is particularly expanded in the Brassicaceae relative to other tested plants. While *E. salsugineum* only has two copies, *A. lyrata* has five copies and even six copies are present *A. thaliana* and *C. rubella* genomes. They are organized in three clades: a VIM2/3/4 clade, a VIM1/5 clade and a VIM6 clade (Figure 3a). In Arabidopsis, redundancy between VIM genes from different clades was observed. A reduction of CG methylation similar to the one observed in *met1* mutant was obtained only in the triple *vim1*;*vim2*;*vim3* mutants and not in single *vim* mutants [9,58,59]. RNA-seq analysis further showed an upregulation of a similar set of genes between *met1* and *vim1*;*vim2*;*vim3* mutants [72]. Altogether, these data suggest that VIM1, VIM2 and VIM3 proteins in Arabidopsis are the main contributors in maintaining CG methylation, potentially by recruiting MET1 as demonstrated for animal counterparts. Unexpectedly, VIM5 ubiquitin ligase activity targets MET1 for degradation rather than to recruit this enzyme to methylated sequences [64]. As no data are currently available for VIM from any other plants, it is unclear whether such novel function is present outside Arabidopsis.

Similarly to VIM proteins, at least one DNA methyltransferase homolog to DNMT1 is detected in all the selected species of algae and land plants (Figure 3b) [56]. Although only one MET copy can be detected in some species*,* the MET gene family has generally expanded in land plants. In Brassicaceae, three MET homologs are detected in *Capsella rubella* or *Eutrema salsugineum* and up to four in *Arabidopsis thaliana* (Figure 3b). Interestingly, they are divided in two separate groups: a MET1 group and a MET2/3 group, suggesting diverging function. Akin to mammals, knock-out mutants in the single *MET* gene in early land plants such as *Marchantia* or *Physcomitrella*, display a genomewide demethylation and pleiotropic developmental phenotypes [73,74]. In rice, mutations in each *MET* gene (*MET1a*, *MET1b*) lead to methylation pattern defects but only *met1b* generated a marked developmental defect [75,76]. This suggests that duplication of rice *MET* genes could have led to the emergence of a distinct non-overlapping function during development. The reason why the MET gene family expanded through evolution and why angiosperm plants are maintaining several copies of potentially functional methyltransferases remains unknown.

Altogether these phylogenetic relationships suggest that homologues of DNMT1 and UHRF1 are present in plant genomes surveyed displaying CG methylation and have been duplicated in some species during plant evolution. Much is still to be done to determine if these novel MET and VIM proteins are devoted to CG methylation maintenance or have evolved other specific functions.

## 5. Alternative MET/VIM Pathways during Reproduction

In flowering plants such as the model plant Arabidopsis, reproduction is initiated late in development when the flower generates organs producing the gametes after two successive phases [77]. During the first phase, called sporogenesis, a diploid germline precursor is selected to undergo meiosis and form the germ cells. During gametogenesis, the male and female germ cells undergo several mitoses to generate, respectively, two male gametes within a vegetative cell in the pollen grain and two female gametes (the egg cell and the central cell) and accessory cells in embryo embedded in the ovule. Upon fertilization, one sperm cell fuses with the egg cell, the second with the central cell to generate, respectively the embryo and the endosperm in a seed. The endosperm is a transient tissue supporting the growth of the embryo akin to the mammalian placenta. In contrast to the embryo, the endosperm does not contribute to the next generation [7,78].

Although DNA methylation patterns are mostly stable over many generations in plants [79,80,81,82], genome-wide DNA methylation profiling of reproductive cells (male meiocytes, sperm, egg and central cells) and fertilization products (embryo, endosperm) revealed highly dynamic DNA methylation patterns in reproductive tissues [83,84,85,86,87,88,89,90].

A genome-wide DNA demethylation was detected in isolated central cells, mainly at non-CG sequences in Arabidopsis [91] due to an active demethylation by the DNA demethylase *DEMETER* (*DME*) expressed in the central cell but barely in the endosperm [29,92]. A passive demethylation was proposed to contribute to this hypomethylated state as the main methyltransferase MET1 is downregulated before central cell differentiation [93,94]. However, expected CG hypomethylation in DME-independent target sequences was not observed, suggesting that the hypomethylated central cell genome only results from an active demethylation process [91]. As *MET1*—but not *DME*—remains expressed in the sperm cells [92,93], parental genomes are differentially methylated in the young endosperm. This asymmetry in DNA methylation can lead to a biased expression of genes depending on their parental origin, which corresponds to a phenomenon called imprinting also encountered in the mammalian placenta [7,95]. After fertilization, the initiated demethylation in the central cell is amplified in the endosperm at non-CG sequences but only slightly affected at CG sequences [91].

Interestingly, the three homologs of *MET1* in Arabidopsis (*MET2a*, *MET2b*, *MET3*) are expressed in cell types where the main methyltransferase *MET1* is not. *MET2a* and *MET2b* are detected in the central cell while *MET3* is detected in the endosperm [94]. These three proteins could constitute alternative CG methylation maintenance pathways during sexual reproduction and potentially influence gene imprinting or seed development. Although the activity of MET1 homologs has not been assessed yet, *met2a* mutant has a limited reduction of methylation at selected transposons [96] and a *met3* mutant called *MATERNAL EFFECT EMBRYO ARREST 57* (*MEE57*) shows an arrest in endosperm development [97]. Expression data for MET proteins of other plants seem to suggest that duplicated *MET* genes also share a complementary expression pattern. In wheat, the nine *MET1-like* genes are members of three paralogous groups: MET2 (a, b and d), MET5 (a, b and d) and MET7 (a, b and d) [60]. Genes of MET2 group are enriched in vegetative tissues while genes of MET5 and MET7 are, respectively expressed in grains and reproductive tissues [60]. In *Brassica rapa*, *BrMET1α* is broadly expressed during plant development, while *BrMET1β* is only expressed in pistils [98].

On the other hand, the expression pattern of the VIM gene family in plants is very limited and restricted to Arabidopsis. The three canonical genes *VIM1*, *2* and *3* are expressed at least during the vegetative phase [65] and *VIM5* is specifically expressed in Arabidopsis endosperm [99]. Further investigations are needed to clarify the contribution of the different MET/VIM proteins to CG methylation maintenance and understand why the MET/VIM gene family diversified during plant evolution but not in mammals.

## 6. Conclusions and Perspectives

Tremendous efforts have been concentrated towards the elucidation of the pathways contributing to non-CG methylation in plants and revealed that they differ from those acting in mammals. In contrast, the pathways maintaining CG methylation in plants remain poorly understood although the core players of CG methylation maintenance DNMT1/MET and UHRF1/VIM are well-conserved between mammals and plants, and that several distinct molecular mechanisms are now determined in mammals. Interestingly, the MET and VIM gene families have diversified during land plant evolution compared to the animal kingdom. The consequences of such an evolutionary trend that offer the potential for functional diversification in CG methylation pathways remain to be explored. This knowledge should also bring insights into whether the differences between the life cycles and lifestyles of animals and plants were key drivers towards the diversification of CG methylation machinery in plants.

## Data Availability

Not applicable.

## Supplementary Materials

Supplementary Materials are available online at www.mdpi.com/article/10.3390/epigenomes5030019/s1.
