# Supplementary material for: Evolution of CG Methylation Maintenance Machinery in Plants
Source: Epigenomes. 2021 Sep 14;5(3):19. doi: 10.3390/epigenomes5030019 (PMC8594673; doi:10.3390/epigenomes5030019)
Supplement: Supplementary file 1 [file epigenomes-05-00019-s001.zip › epigenomes-1326197-supplementary final.pdf]

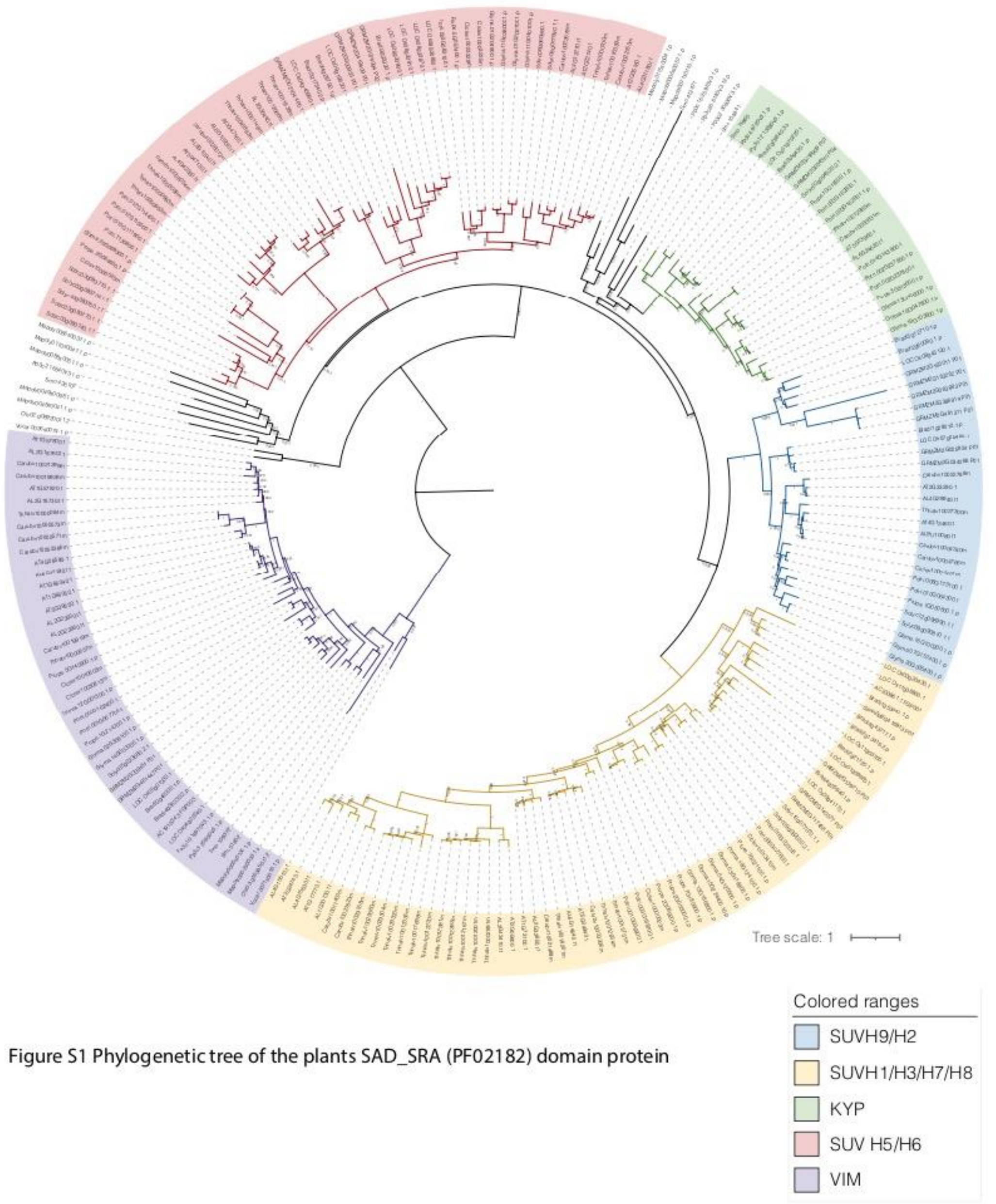

Figure S1. Circular phylogram of plant SAD_SRA (PF02182) domain proteins. The different clades are colored as indicated in the associated legend. The bootstrap number is represented on each branch. SAD_SRA (PF02182) domain protein sequences were downloaded from Phytozome [71] with a Pfam domain filter (SAD_SRA, PF02182). We restricted our analysis to the following representative species: *A. lyrata*, *A. thaliana*, *B. distachyon*, *C. rubella*, *C. reinhardtii*, *C. clementine*, *E. salsugineum*, *G. max*, *M. polymorpha*, *O. sativa*, *P. patens*, *P. trichocarpa*, *P. persica*, *S. moellendorfii*, *S. lycopersicum*, *V. carteri*, *Z. mays*. The sequences were cleaned using CD-HIT suite with a sequence identity cut-off of 1 and length of the sequence to skip 300 [100]and further manually curated to remove protein variants (i.e., only one representative protein per locus). Alignment and phylogenetic reconstructions were performed using the function "build" of ETE3 v3.0.0b32 [101] as implemented on the GenomeNet (https://www.genome.jp/tools/ete/, accessed on 18 July 2021) using default parameters. The mid-point rooted tree was exported in a Newick format. Vizualisation and illustration were performed using iTOL v6.1.2 [102].

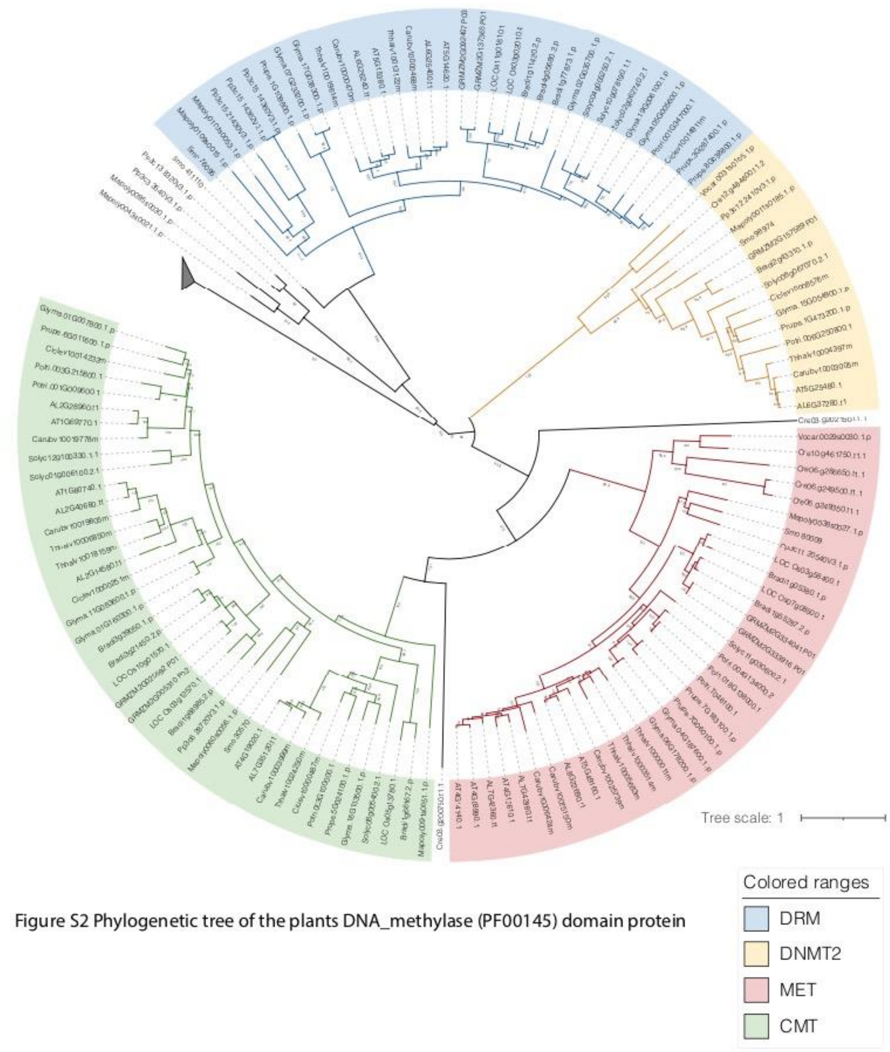

Figure S2. Circular phylogram of plant DNA methylase (PF00145) domain proteins. The different clades are colored as indicated in the associated legend. The bootstrap number is represented on each branch. DNA methylase (PF00145) domain protein sequences were downloaded from Phytozome [71] with a Pfam domain filter (DNA methylase, PF00145). We restricted our analysis to the following representative species: *A. lyrata*, *A. thaliana*, *B. distachyon*, *C. rubella*, *C. reinhardtii*, *C. clementine*, *E. salsugineum*, *G. max*, *M. polymorpha*, *O. sativa*, *P. patens*, *P. trichocarpa*, *P. persica*, *S. moellendorfii*, *S. lycopersicum*, *V. carteri, Z. mays*. The sequences were cleaned using CD-HIT with a sequence identity cut-off of 1 and length of the sequence to skip 300 [100] and further manually curated to remove protein variants (i.e., only one representative protein per locus). Alignment and phylogenetic reconstructions were performed using the function "build" of ETE3 v3.0.0b32 [101] as implemented on the GenomeNet (https://www.genome.jp/tools/ete/, accessed on 18 July 2021) using default parameters. The mid-point rooted tree was exported in a Newick format. Vizualisation and illustration were performed using iTOL v6.1.2 [102]
